# Fracture Resistance of 3-Unit Zirconia Fixed Dental Prostheses Differing in Wall Thickness Fabricated by Either 3D-Printing or Milling

**DOI:** 10.3390/jfb16090330

**Published:** 2025-09-05

**Authors:** Stefan Rues, Jannis Crocoll, Sebastian Hetzler, Johannes Rossipal, Peter Rammelsberg, Andreas Zenthöfer

**Affiliations:** Department of Prosthodontics, Medical Faculty, Heidelberg University, 69120 Heidelberg, Germany; jannis.crocoll@stud.uni-heidelberg.de (J.C.); johannes.rossipal@med.uni-heidelberg.de (J.R.); peter.rammelsberg@med.uni-heidelberg.de (P.R.); andreas.zenthoefer@med.uni-heidelberg.de (A.Z.)

**Keywords:** zirconia, 3D zirconia printing, 3-unit FDP, individual printing parameters, artificial aging, oblique loading, fracture resistance

## Abstract

Background: To evaluate the fracture resistance of 3D-printed 3-unit fixed dental prostheses (FDPs) made from tetragonal zirconia polycrystal (3Y-TZP). Methods: Based on a maxillary typodont model with a missing first molar and neighboring teeth with full crown preparations, FDPs differing in wall thickness (d = 0.6 mm / d = 0.8 mm / d = 1.0 mm) were designed. For all test groups, 12 samples were fabricated from 3Y-TZP by either 3D-printing or milling. For 3D-printing, pontic designs were modified by basal slots to enable regular firing times. After luting on CoCr dies, samples underwent artificial aging. Loads tilted by 30° were applied on the mesio-buccal cusp of the pontic, and fracture resistance F_u_ was assessed. Welch ANOVA and Dunnett-T3 tests were used for statistical evaluation. Results: Significant differences in F_u_ were identified (Welch ANOVA, *p* < 0.001). For milled FDPs, fracture originated from connector areas, and F_u_ increased with increasing wall thickness (d = 0.6 mm: 1536 ± 131 N, d = 0.8 mm: 2226 ± 145 N, d = 1.0 mm: 2686 ± 127 N, significant differences but for the comparison d = 0.8 mm vs. d = 1.0 mm). For 3D-printed FDPs, the loaded cusp fractured, and F_u_ did not change with FDP wall thicknesses (*p* > 0.779, F_u_ = 1110 ± 26 N for all PZ FDPs). Milled FDPs showed significantly higher F_u_ when compared to 3D-printed FDPs with identical wall thickness. Conclusions: Although 3D-printed zirconia FDPs still show lower fracture resistance values than their milled counterparts, all tested FDP configurations clearly exceed the clinical reference thresholds and can therefore be recommended for clinical use.

## 1. Introduction

Conventional end-abutment fixed dental prostheses (FDPs) supply a common treatment option for the rehabilitation of edentulous spaces. The desire of patients for tooth-colored materials has fueled the use of all-ceramic materials. FDP frameworks are frequently made of zirconia and are veneered with glass ceramics [1,2]. Despite some optimizations like long-term cooling [3] and anatomical framework design, veneer fractures still occur commonly [1,4]. In the last decade, zirconia has gained further importance, as it can nowadays be used for monolithic restorations without additional veneering, at least in the posterior tooth regions. The classic fabrication approach for zirconia restorations is the CAD/CAM (computer-aided design/computer-aided manufacturing) workflow by aid of milling technology. The milling process is well studied and provides reliable and well-fitting crowns and FDPs [5]. Disadvantages are limitations in the geometric CAD design as well as so-called tooling stress, which can lead to material bursting, for example, in the case of thin restoration edges. To compensate for these disadvantages, CAM strategies have been further developed, in particular, for the manufacturing of defect-oriented minimally invasive prostheses.

The additive manufacturing of zirconia by means of 3D-printing is novel and has the potential to overcome the described immanent limitations of the milling technology. For instance, structures can be fabricated as designed, and—theoretically—waste and time can be reduced [6,7]. With digital light processing (DLP) or stereolithography (SLA), refined suspensions consisting of light-curing acrylate binder and zirconia filings provide the substrate for these efforts. After 3D-printing, the objects are cleaned of excess non-cured material with solvents like isopropanol and subsequently subjected to heat treatment to remove the resin component (debinding) before fully sintering the material to its final density. Previous studies by several research groups [8,9,10] indicated that 3D-printed zirconia clearly exceeded the ISO 6872 [11] threshold for flexural strength of 500 MPa specified for 3-unit FDPs in the molar region, although 3D-printed zirconia is more prone to structural defects compared to milled zirconia. Most information is given for the strongest zirconia type, i.e., tetragonal zirconia polycrystal materials doped with 3 mol% yttria (3Y-TZP), since this material was available for 3D-printing beginning with initial market introduction. Especially with the goal of a clinical use of this new technology, it is to be recommended to start with this material, providing the smallest risk of technical complications. Stabilizing the cubic phase next to the tetragonal phase by increasing the yttria content to 4 or 5 mol% leads to more esthetic but less strong partially stabilized zirconia materials (4Y/5Y-PSZ). Up to now, 3D-printed zirconia materials often show increased microporosity [12,13] compared to milled zirconia, leading to anisotropic behavior (tensile strength higher for stresses acting in the horizontal plane compared to stresses acting in the vertical layering direction) [14] and inferior esthetics.

Besides the properties mentioned above, 3D-printed zirconia materials exhibit other promising material parameters, including the absence of cytotoxicity and good biocompatibility [15]. These parameters, as well as in vitro studies reporting clinically acceptable results with regard to fit and fracture load of 3D-printed zirconia veneers or crowns [16,17], warrant further investigation of larger restorations such as 3-unit FDPs. In contrast to single-unit restorations, fabrication of, i.e., 3-unit zirconia FDPs by 3D-printing is much more challenging, and the few studies on this topic to date highlight that accuracy and fit [18] as well as fracture resistance [19] on par with the milling technology are not yet given. In particular, the massive pontics of FDPs in the posterior region may suffer from material degradation or even crack formation during firing. This is why most manufacturers provide several firing protocols, increasing total firing time the thicker the 3D-printed objects become. For a 3-unit FDP in the molar region with a massive pontic, recommended firing time would be almost a week with the 3D-printer and material used in this investigation. Such long fabrication times compared to those of milled restorations with overnight sintering or even speed-sintering in less than an hour for crowns or 3-unit FDPs would not be accepted by dental technicians unless there was no alternative. To circumvent this problem, the wall thickness of the pontic could be decreased by creating, for example, a hollow pontic as already performed by other research groups with FDPs printed from composite materials [20].

When loaded up to failure, connectors of minimally invasive 3-unit FDPs with cross-sections > 9 mm^2^ do in general not state the weakest link but the transition area where the connector meets the rather thin wall of the abutment crown [21]. When analyzing fracture surfaces of such FDPs, typically a part of the abutment crown surface is missing. As a consequence, wall thickness can—as long as the connector transition area is the highest stressed region—be expected to have a tremendous effect on the FDP’s fracture resistance [22]. For zirconia materials processed by milling, manufacturers provide clear guidelines for specific indications. In particular, minimum wall thickness for monolithic 3Y-TZP restoration is typically specified with 0.5–0.6 mm.

Manufacturing strategies and materials science tests for the performance of (minimally invasive) 3D-printed zirconia FDPs are missing. To the best knowledge of the authors, this is the first systematic study investigating the fracture resistance of 3D-printed zirconia FDPs. The aim of the current study was to investigate the fracture resistance of 3Y-TZP 3-unit FDPs produced by means of 3D-printing and milling with different wall thicknesses to be able to give recommendations on minimum wall thicknesses for clinical use. The null hypotheses were that neither wall thickness (1) nor fabrication technology (2) would affect the fracture resistance of the zirconia FDPs.

## 2. Materials and Methods

### 2.1. Individual Calibration of 3D-Printing Parameters

**Figure 1:**
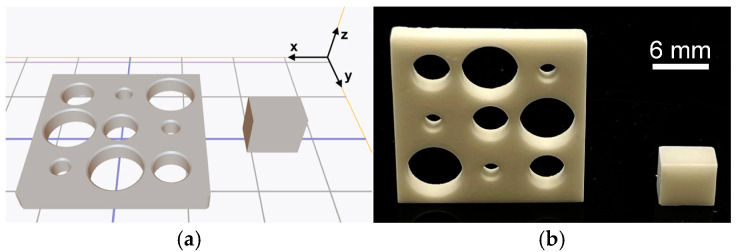
Calibration objects, (**a**) screenshot showing the nesting orientation of the scaled objects (10 mm grid distance), and (**b**) final objects after debinding and sintering.

The larger the printed object, the higher the need for accurate printing parameters such as scaling factors and xy-offset $O_{xy}$ to compensate for overpolymerization along the boundaries of areas exposed to light during curing. In general, manufacturers of 3D-printers just provide universal scaling factors $S_{x,univ},S_{y,univ}{,\;\;and\;S}_{z,univ}$ and little information about compensation of overpolymerization. Therefore, an individual determination of these parameters ($S_{x,indiv},S_{y,indiv}{,\;S}_{z,indiv}{,\;\;and\;O}_{xy}$) was realized at regular intervals and for each new batch of zirconia printing material as follows:

For the assessment of ${\;S}_{z,indiv}$, six blocks with final dimensions of 6 mm × 4 mm × 5 mm were printed using the universal scaling factors. To determine the remaining parameters, an xy-calibration object was created as follows: in a disc with dimensions 16 mm × 16 mm × 2 mm, nine cylindrical holes (index *i*) were added with center points arranged in a grid with a nominal distance $d_{nom}$ = 6 mm. The cylindrical holes had nominal radii ${\;r}_{i}$ of either 1 mm, 2 mm, or 3 mm. Before printing three xy-calibration objects, scaling took place with universal scaling factors, and no xy-offset was implemented. Figure 1a shows the nested CAD files, whereas in Figure 1b the final zirconia objects can be seen.

**Figure 2:**
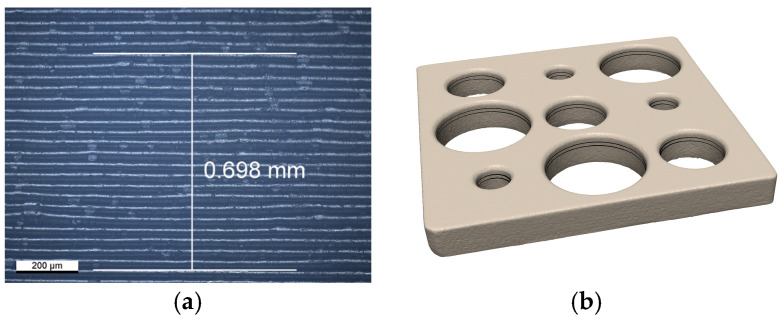
(**a**) Image taken with a digital microscope at 300× magnification from a z-calibration object to measure the height of 20 printed layers, and (**b**) 3D-scan of a xy-calibration object with horizontal cross sections at 0.5 mm and 0.7 mm distance from the upper surface.

Postprocessing of the printed objects was identical to the procedure later described for the FDPs. Along the central axis of the largest area of each z-calibration object, the vertical dimension of 20 printed layers was measured at 3 different heights with a digital microscope (Smartzoom 5; Carl Zeiss AG, Jena, Germany) at 300× magnification (Figure 2a). Using a layer thickness of 50 µm, the nominal height in the printed state was $h_{nom,print}$ = 20∙50 µm = 1 mm. Together with the corresponding mean height $h_{final,mean}$ gained by averaging the 18 measured heights for 20 layers found in the final z-calibration objects, the individual vertical scaling factor was given by ${\;S}_{z,indiv}=\;h_{nom,print}/h_{final,mean}$.

If shrinkage for the xy-calibration objects differs in x- and y-direction and the universal scaling parameters are not exact, the cross-sections of the cylindrical holes will have an elliptic shape for the final objects, which can be mathematically described as given in (1). With a given ratio $k_{y}$ of half axes a and b, this equation can be transformed (2).

(1) $$\frac{x^{2}}{a^{2}}+\frac{y^{2}}{b^{2}}=1$$

(2) $$\sqrt{x^{2}+\frac{y^{2}}{k_{y}^{2}}}=a\;\;\;with\;b=k_{y}a$$

(3) $$\begin{array}{c} {f=\sum_{i=1}^{9}\sum_{j=1}^{n_{i}}\left(\sqrt{{\left(x_{i,j}-x_{c,i}\right)}^{2}+\frac{{\left(y_{i,j}-y_{c,i}\right)}^{2}}{k_{y}^{2}}}-a_{i}\right)}^{2} \end{array}$$

with the following:

*i*: number of the respective cylindrical opening;
*n_i_*: number of points describing the contour line of the *i*th cylindrical opening at a given height z;
(x_c,i_, y_c,i_): center point coordinates of an ellipse fitted to the cylindrical opening *i*;
a_i_: first half-axis of an ellipse fitted to the *i*th opening;
k_y_: half-axis ratio (constant for all cylindrical openings).

Final xy-calibration objects were sandblasted (50 µm alumina particles, 0.2 MPa) and digitized (D2000 and Convince 2015; 3shape, Copenhagen, Denmark) with a scan sequence consisting of 24 single scans (50 µm resolution, 20° or 50° angulation with respect to the horizontal plane, 12 equally distributed circumferential scan positions). The surface was triangulated with a homogeneous mesh with 30 µm triangle edge length, and data was exported as stl (standard tessellation language) files. After importing the surface data in CAD software (Geomagic Design X V2024.3.0; 3D Systems Inc., Rock Hill, SC, USA), a plane was fitted to the upper surface of the scanned xy-calibration object. Then the object was rotated such that the normal vector of the fitted plane was oriented exactly in the z-direction. Two parallel planes at heights Δz = −0.5 mm and Δz = −0.7 mm below the surface and splines defined by cutting the stl-surface with the respective plane were created. The chosen accuracy for the splines describing the cross-sections of the cylindrical opening was <1 µm (Figure 2b). For the following optimization, about 360 equidistant points were defined on each spline, and the data were exported.

Best-fitting ellipses with a constant half-axis ratio were determined separately for cross sections generated at the two different heights by minimizing the objective function given in (3) using the Matlab Optimization toolbox (Matlab 2022b; Mathworks Inc., Natick, MA, USA). The objective function consisted of 28 variables, i.e., center point coordinates and first half axes of all nine cylindrical openings as well as the half axis ratio.

Since an overpolymerization during 3D-printing will not change the center points of the cylindrical openings, the scaling factors can be computed without knowledge about the size of this effect. After calculating the six distances between neighboring center points in each x- and y-direction, their mean values $d_{x,mean}$ and $d_{y,mean}$ can used to calculate the individual scaling factors as presented in (4) and (5). Individual scaling factors were gained by averaging the results of the two cross sections at different heights for all three printed xy-calibration objects.

(4) $$S_{x,indiv}=\frac{d_{nom}}{d_{x,mean}}S_{x,univ}$$

(5) $$S_{y,indiv}=\frac{d_{nom}}{d_{y,mean}}S_{y,univ}$$

**Figure 3:**
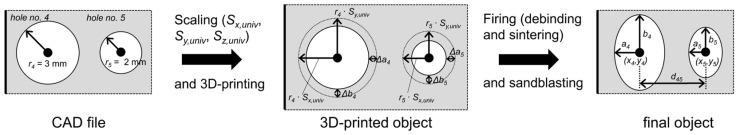
Scheme showing relevant measures in an xy-cross-section for 3D-printed xy-calibration objects, exemplarily for two (holes 4 and 5) of the nine holes arranged in a 6 mm grid.

The overpolymerization takes place during printing, i.e., in the scaled state. As shown in Figure 3, the xy-calibration object was scaled using the universal scaling factors. The overpolymerization caused a reduction of the hole diameter (dimensional changes $∆a_{i}$ and $∆b_{i}$), whereas subsequent firing caused a shrinkage described by the individual scaling factor, sresulting in openings in the final state with half axes $a_{i}$ and $b_{i}$. Thus, the overpolymerization can be computed as follows (Equations (6) and (7)) for any cylindrical opening (index *i*):

(6) $$\frac{\left(r_{i}\cdot S_{x,univ}-∆a_{i}\right)}{S_{x,indiv}}=a_{i}\;\;\to \;\;\;∆a_{i}=r_{i}\cdot S_{x,univ}-a_{i}\cdot S_{x,indiv}$$

(7) $$\frac{\left(r_{i}\cdot S_{y,univ}-∆b_{i}\right)}{S_{y,indiv}}=b_{i}\;\;\to \;\;\;∆b_{i}=r_{i}\cdot S_{y,univ}-b_{i}\cdot S_{y,indiv}$$

Ideally, the calculated overpolymerization is a constant value and does not depend on the size of the cylindrical opening. To be able to check this, different hole diameters were implemented in the xy-calibration object. The offset $O_{xy,indiv}$ is meant to compensate for the overpolymerization and has therefore the opposite sign. Using data of both cross sections and cylindrical openings, this last parameter can be determined (Equation (8)) for each xy-calibration object, and a final mean value over all objects can be computed.

(8) $$O_{xy,indiv}=-\frac{1}{36}\sum_{z=1}^{2}\sum_{i=1}^{9}\left(∆a_{i,z}+∆b_{i,z}\right)$$

### 2.2. Study Design and Sample Fabrication

In this in vitro study, six FDP test groups were compared, differing in CAM fabrication (3D-printed vs. milled) and FDP wall thickness (d = 0.6 mm / 0.8 mm / 1.0 mm). At the time this study was planned, no data existed on the fracture resistance of minimally invasive 3D-printed FDPs in the posterior region. Therefore, this was an explorative study. Based on a previous investigation with 3D-printed occlusal veneers differing in wall thickness, for which a sample size of n = 8 sufficed to identify significant group differences [23], an even higher sample size of n = 12 was chosen for fracture load testing of 3-unit FDPs.

**Figure 4:**
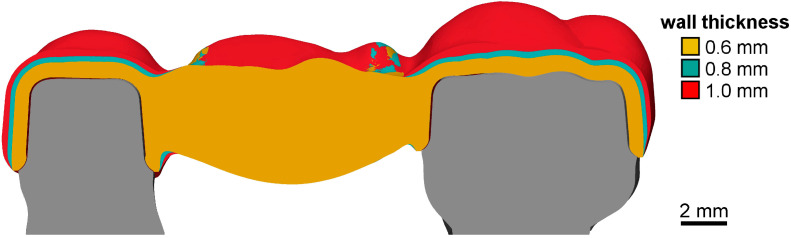
Cross section through the three different FDP designs with 0.6 mm (orange), 0.8 mm (cyan), or 1.0 mm (yellow) abutment crown wall thickness.

In a typodont upper jaw model (ANA-01; Frasaco GmbH, Tettnang, Germany) with a missing first molar, second premolar, and second molar, they were prepared with a unilateral angle of 4° and a chamfer design along the finishing line. After model digitization (D2000; 3shape), a fixed dental prosthesis (FDP) with 0.6 mm wall thickness, 30 µm marginal gap width, and 80 µm cement gap width was designed (Dental Designer 2018; 3shape). Twice, the design job was copied, reset, and repeated using the existing design steps and only changing wall thickness to either 0.8 mm or 1.0 mm, thus creating FDPs with identical margin lines and pontic geometry, identical connectors, but differing abutment crown wall thickness (Figure 4). For all designed restorations, the mesio-palatinal cusp of the pontic was modified (Geomagic Design X V2024.3.0; 3D Systems Inc.) to show a constant tilt of 30° with respect to the horizontal plane in the buccal direction.

Dies for fracture load testing consisted of the respective abutment crown geometry complemented with standardized conical roots with a rectangular cross-section. Dies for the molar as well as premolar abutment tooth were then 3D-printed, each 36 times, from burnable resin (Freeprint Cast; Detax GmbH, Ettlingen, Germany), cleaned, and finally cast with a cobalt-chromium (CoCr) alloy (Remanium 2000; Dentaurum GmbH & Co. KG, Ispringen, Germany).

**Figure 5:**
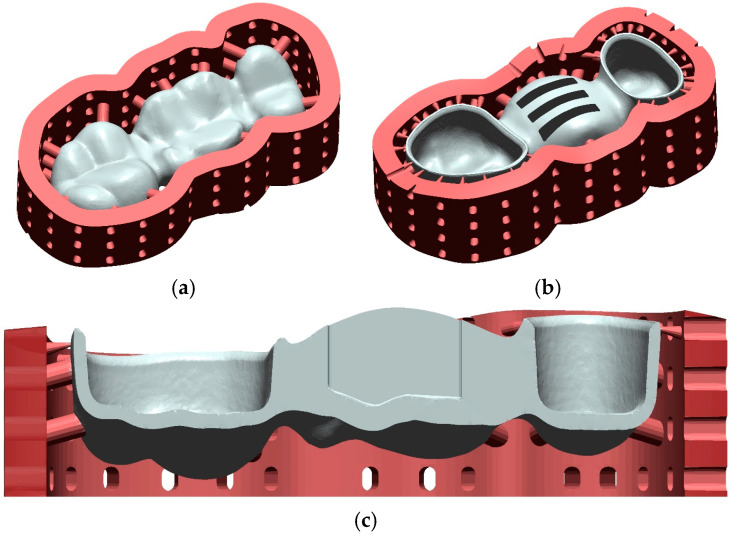
Designed FDP (white) with support structures and sintering frame (red) for 3D zirconia printing. (**a**) occlusal view, (**b**) basal view showing the three added slots in the pontic, and (**c**) vertical cross-section through the FDP along the mesio-distal axis.

**Figure 6:**
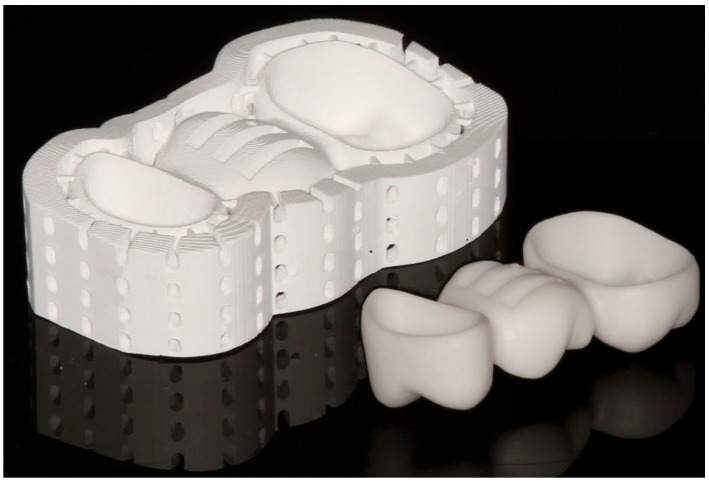
Three-dimensional-printed zirconia FDPs after cleaning as well as after final firing.

Each FDP design was fabricated 12 times from tetragonal zirconia polycrystal doped with 3 mol% yttria (3Y-TZP) using either milling technology (Cercon ht/Cercon Brain Expert; Dentsply Sirona Inc., Hanau, Germany) or 3D-printing (InniCera BCM/ZIPRO; AON Co., Ltd., Seoul, South Korea). To avoid any misfit originating from inaccuracies during die replication, dies had been individually scanned (D2000; 3shape) and aligned with the reference model situation (Geomagic Design X V2024.3.0; 3D Systems Inc.) in advance, and the individual crown cavities with identical cement gap settings as described above (Dental Designer; 3shape) replaced those of the standard design while all other surface aspects remained unchanged (Geomagic Design X V2024.3.0; 3D Systems Inc.). Milled FDPs were sintered for 8 h at temperatures up to 1500 °C (Cercon heat plus; Dentsply Sirona Inc.) according to the manufacturer’s instructions. For the printed FDPs, preprocessing consisted of several steps. After choosing the FDP nesting orientation, scaling and xy-offset correction with the previously assessed parameters was performed with a self-programmed function (Matlab 2022b; Mathworks Inc.) by changing the coordinates of the vertices of the FDPs stl-surfaces but keeping the original triangulation. Subsequently, a stiffening frame had to be added (Figure 5a–c) to reduce distortions during the firing procedure consisting of debinding and sintering. Since massive pontics would necessitate very long firing times to omit material degradation or fractures during fabrication, FDP pontics were modified with 3 slots (8 mm length, 1.5 mm width, 1.4 mm distance between the slots) from the basal side (Figure 5b), enabling a rather short firing protocol. The slots stopped at 1 mm distance from the occlusal surface in the scaled model, i.e., at about 0.75 mm distance in the fully sintered state (Figure 5c), and internal slot edges were rounded. Data was then transferred to the printer’s preprocessing software (ZiproS V3.1.106; AON Co., Ltd.), and slicing was performed with 50 µm layer thickness. Three-dimensional printing took place with parameters predefined by the manufacturer, and the temperature during printing was kept between 26 °C and 28 °C. During postprocessing, occlusal support structures were removed from printed FDPs, and surfaces were cleaned from excess slurry with an airbrush at 0.1 MPa and isopropanol as a solvent. After the first firing (Table 1) for 30 h at temperatures up to 1100 °C (ZIRFUR, AON Co., Ltd.), the sintering frame could be easily removed in the presintered state. In a clinical workflow, individual staining would be possible, but this step was omitted in this investigation. Finally, FDPs were fully sintered for about 7:20 h (Table 2) at temperatures up to 1500 °C (SINTRA PRO/120zrf; Shenpaz Dental Ltd., Migdal HaEmek, Israel). Besides sandblasting of the surfaces, no polishing or glazing of the FDPs was carried out in this investigation. Exemplary FDPs before and after firing are displayed in Figure 6. Basal slot surfaces were tribochemically preconditioned (Rocatec Pre/Plus; Solventum GmbH, Kamen, Germany; air pressure: 0.28 MPa), coated with MDP-containing primer (Clearfill Ceramic Primer Plus; Kuraray Co., Ltd., Tokyo, Japan), and the slots were filled with dual-curing composite resin (Rebilda DC; VOCO GmbH, Cuxhaven, Germany).

Before cementation and mechanical loading of the samples, individual models were fabricated. After coating the root sections of the dies with heat-shrink tubings (8620120956; DSG Canusa GmbH, Rheinbach, Germany) and closing the apical opening of the tubings with silicone (Flexitime Correct Flow; Kulzer GmbH, Hanau, Germany), CoCr abutments were inserted into the respective FDP and provisionally fixed. With the help of a paralleling device and a mold having the negative shape of the pontic’s occlusal surface, the abutment teeth—together with the FDP—were lowered in aluminum blocks and embedded with acrylic resin (Technovit 4071; Kulzer GmbH). With this setup, the physiological resilience of natural teeth (0.1 mm insertion for 100 N centric axial loading and 0.3–0.4 mm deflection for application of 100 N horizontal force on the occlusal level) could be simulated. After finishing the curing of the acrylic resin, the FDP was removed, and both the FDP and CoCr teeth were steam cleaned. CoCr teeth were sandblasted (110 µm alumina particles, 0.1 MPa), whereas inner FDP surfaces were tribochemically preconditioned and primed as described above for the basal slots. Finally, FDPs were attached to their abutment models by use of a composite cement (Panavia 21; Kuraray Co., Ltd.) under application of a constant load of 200 N in a universal testing device (Z005; Zwick/Roell AG, Ulm, Germany). After cementation, samples were stored at 100% humidity and 37 °C for 1 d.

### 2.3. Artificial Aging and Fracture Load Tests

**Figure 7:**
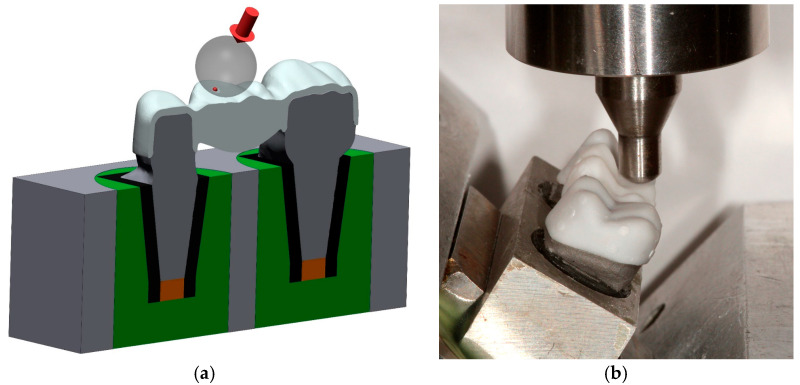
Test setup for FDP fracture load testing. (**a**) Scheme showing a cross section of the setup with the FDP placed on CoCr abutment teeth. To simulate clinical tooth mobility, standardized root sections were covered with heat-shrink tubing before fixation in an aluminum mold with acrylic resin. Forces were applied to the mesio-buccal cusp with a steel sphere. (**b**) Test setup in the universal testing device. The FDP was tilted by 30° such that the loaded cusp was oriented horizontally.

All samples were subjected to artificial aging, including 1.2 million cycles of chewing simulation (CS 4.8; SD Mechatronik GmbH, Feldkirchen, Germany) with a force magnitude of 108 N and 30 days of water storage at 37 °C. During mechanical loading, samples were tilted by 30° such that the loaded cusp was oriented horizontally (Figure 7a,b) and the load application site was at a 2 mm distance from the tip of the cusp. Samples were visually inspected for flaws or cracks before and after artificial aging (Smartzoom 5; Carl Zeiss AG). Samples surviving chewing simulation were tested for their fracture resistance F_u_ in a universal testing device (Z005; Zwick/Roell AG) with a steel indenter having a spherical tip and a diameter of 6 mm. Fracture tests were conducted with a crosshead speed of 0.5 mm/min and ended when a drop in test force greater than 90% of the maximum test force was registered.

### 2.4. Fractography

Fracture surfaces of all samples were documented with a digital microscope (Smartzoom5; Carl Zeiss AG). For select samples, additional images were taken with an electron microscope (JSM 6510; JEOL Ltd., Akishima, Japan).

### 2.5. Statistical Evaluation

Descriptive statistics comprised mean values/standard deviations (SD) and visualizations by use of box plot diagrams. Group membership with regard to the CAM fabrication approach and wall thickness was evaluated. Since data were normally distributed according to Shapiro–Wilk tests but group variances differed drastically, Welch ANOVA and Dunnett-T3 post hoc tests were used with a significance level of α = 0.05 (SPSS Ver.28, IBM Co., Armonk, NY, USA).

## 3. Results

### 3.1. Individual Calibration

**Figure 8:**
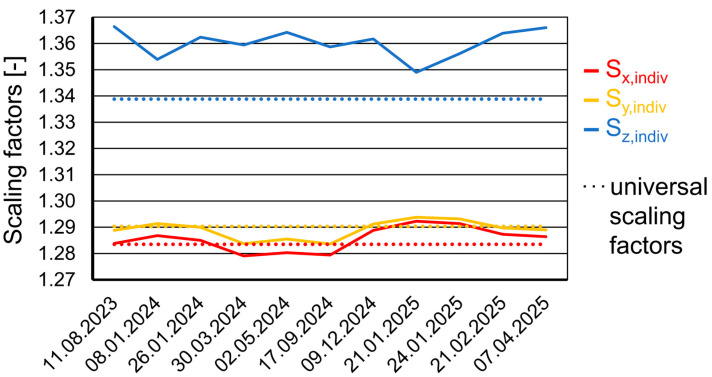
Individual scaling factors were assessed at different dates for the 3Y-TZP material used in this investigation with 3D-printing. The universal scaling factors provided by the manufacturer are displayed as dotted lines.

In Figure 8, calibration results for the 3Y-TZP material InniCera BCM between August 2023 and April 2025 are summarized. The highest deviations between universal and individual scaling factors were given for the vertical direction. As given by the universal scaling parameters, shrinkage in the y-direction was always slightly larger than shrinkage observed for the x-direction. Individual scaling factors were not constant over time. The offset parameter O_xy_ always ranged between −30 µm and −20 µm.

### 3.2. Artificial Aging and Fracure Load Testing

**Figure 9:**
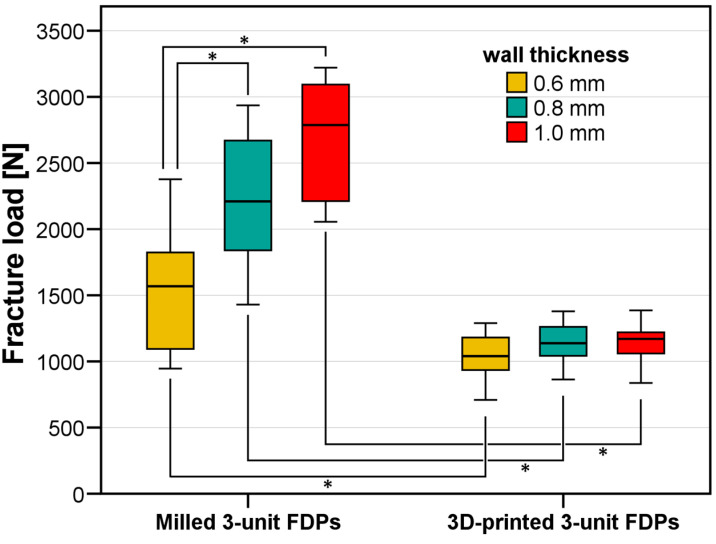
Boxplot diagram showing the fracture loads measured for the different FDP test groups differing in CAM fabrication and wall thickness. Significant differences found by pairwise Dunnett-T3 post-hoc tests are indicated with asterixes.

All FDPs survived artificial aging without macroscopic damage. Regarding the subsequent loading test to fracture, Welch ANOVA identified significant differences among the test groups (*p* < 0.001) with regard to FDP fracture load F_u_ (Figure 9). For the milled FDPs, there was a clear increase in fracture resistance with increasing wall thickness d from F_u_ = 1536 ± 131 N for d = 0.6 mm to F_u_ = 2226 ± 145 N for d = 0.8 mm and F_u_ = 2686 ± 127 N for d = 1.0 mm. Significant differences were observed among the groups (*p* ≤ 0.026), except between the 0.8 mm and 1.0 mm groups, where no significant difference was found (*p* = 0.291). In contrast to the milled FDPs, regardless of wall thickness, a similar FDP fracture resistance of about 1100 N was recorded for all 3D-printed test groups. In detail, fracture resistances were F_u_ = 1043 ± 167 N for d = 0.6 mm, F_u_ = 1138 ± 149 N for d = 0.8 mm, and F_u_ = 1148 ± 145 N for d = 1.0 mm. When comparing milled and 3D-printed FDPs with identical wall thickness, significantly higher fracture resistances were given for the milled FDPs (*p* ≤ 0.043 for all pairwise tests).

### 3.3. Fractograpgy

**Figure 10:**
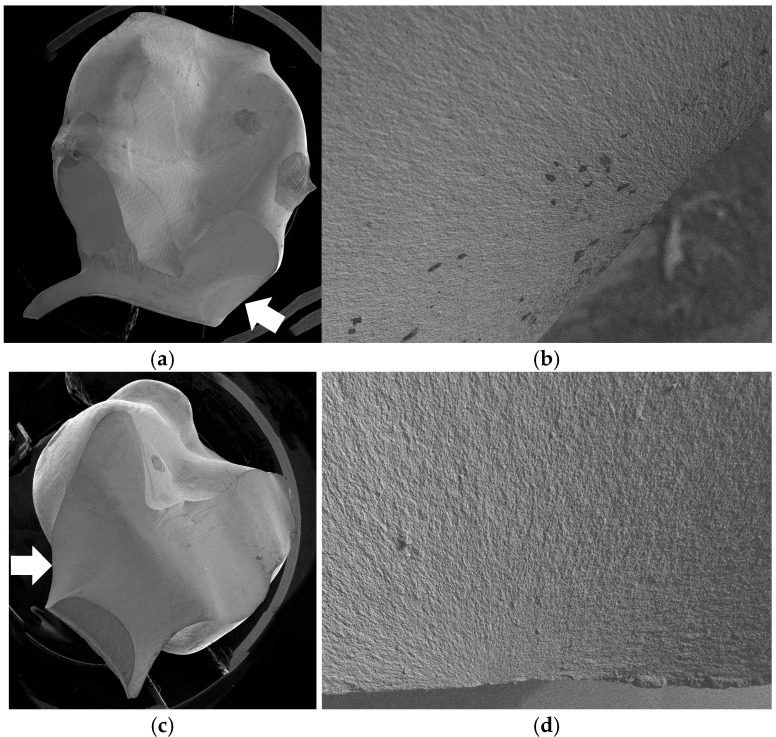
Typical fracture surfaces for milled FDPs. (**a**) Overview of a fracture surface through the distal connector and (**b**) fracture origin at 100× magnitude. (**c**) Overview of a fracture surface through the mesial connector and (**d**) fracture origin at 150× magnitude. In the overview images, white arrows indicate the fracture origin, and the marked load application sites on the mesio-buccal cusps of the pontic are also visible.

For milled FDPs, fracture originated from the mesio-basal surface of the connectors. For thin-walled FDPs with d = 0.6 mm, only one connector fractured in most cases, fractures involving both connectors became more frequent with increasing wall thickness. Figure 10a–d shows typical fracture surfaces of milled FDPs.

**Figure 11:**
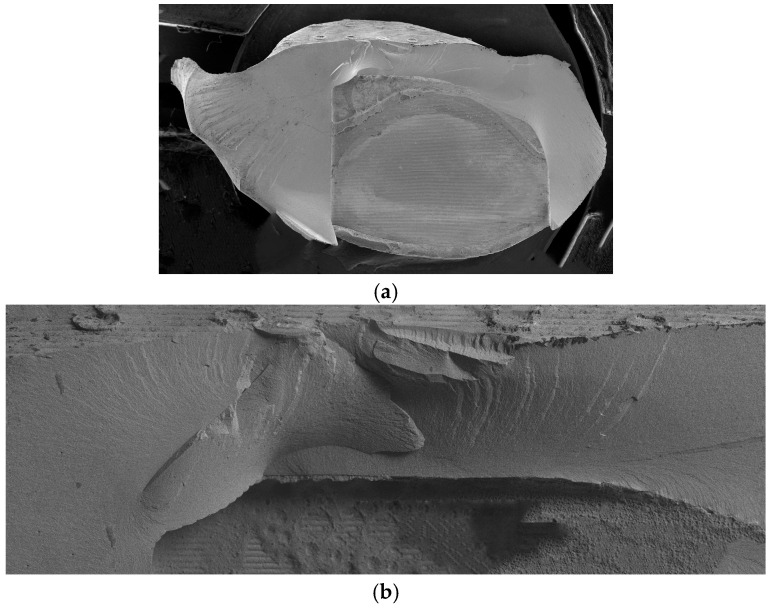
Typical fracture surfaces for a 3D-printed FDP. (**a**) Overview showing the fractured-off loaded cusp and (**b**) a detail of the fracture surface in the vicinity of the load application site.

For 3D-printed FDPs, the loaded cusp fractured, and the fracture originated from the loading site. In almost all cases only the loaded cusp chipped off along a basal slot (Figure 11a,b). Only in a few cases did fracture surfaces propagate to the connector regions.

## 4. Discussion

The first null hypothesis that CAM fabrication technology did not affect fracture resistance had to be rejected, whereas the second null hypothesis that fracture load was not influenced by wall thickness had only to be rejected for 3D-printed FDPs. This outcome was most likely observed due to the geometric modification of the pontic with basal slots for the 3D-printed FDPs. Pontics with slots fractured before critical load levels for the connectors were reached, and since the pontic design was identical for all 3D-printed FDPs irrespective of the wall thickness, similar mean fracture loads of about F_u_ = 1100 N were found for all test groups. Further studies should investigate an optimized hollow pontic design for 3D-printing, enabling an easy cleaning process (removal of excess uncured suspension from the hollow structures) and short fabrication times without material degradation, as well as FDPs with high fracture resistance and acceptable fit. When comparing milled and 3D-printed FDPs with d = 0.6 mm wall thickness, milled specimens could withstand, on average, about 500 N higher forces until they fractured through the connectors. If the pontic design for 3D-printed FDPs was optimized, this gap would be reduced or even closed. With a massive pontic, as seen for the milled specimens, the connector regions were the weakest spot of the restoration even for wall thicknesses of d = 1.0 mm with fracture resistances F_u_ > 2000 N for all specimens of the respective test group. When comparing our results with those of a recent study with 3D-printed 3-unit FDPs also replacing a first molar but using high wall thicknesses between 1 and 2 mm and a massive pontic [19], FDPs fabricated with the same DLP system and zirconia material were inferior in fracture load (F_u_ = 821 ± 160 N), although less critical test conditions were given (axial and centric load application, no simulated tooth resilience). For the other two 3D-printing systems investigated by this research group, similar mean fracture resistances as in our investigation were found. What is also astounding is that despite the massive pontics, shorter firing protocols were used; in particular, 21 h for debinding and presintering up to 1100 °C for the DLP group. In a pilot study with FDPs with massive pontics, our research group had observed a high percentage of FDPs showing major cracks after such a firing with a 30 h duration. However, both approaches reached fracture resistances sufficient for clinical recommendation. While Dagistan et al. suggested a 790 N threshold for their load case [19], our research group uses a 500 N threshold for restorations placed in the posterior region. As all FDP subgroups clearly reached higher mean facture resistances they could unreservedly be recommended for clinical use with 3-unit-FDPs in the posterior region. Beyond the fact that no increase of fracture resistance depending on thicker walls for the 3D-printed FDPs was seen due to the pontic with basal slots being the weakest point, one might also speculate that minor structural flaws and porosity [10] were apparent for the slightly lower fracture resistance. However, with the observed in vitro fracture loads after artificial aging for the critical but clinically relevant load case used in this investigation, a clinical recommendation for these restorations can be given. Limitations of this study were that only one FDP situation and one load case were tested. In real patients, tooth preparations might deviate from the ideal parameters realized for the in vitro model of this investigation. Since 3D-printed zirconia is sensitive to postprocessing, this was performed by one person to exclude any unwanted effects. In a dental lab with many technicians fabricating 3D-printed FDPs, there may be person-specific quality differences. Ultimately, only clinical studies can provide reliable information about in vivo performance.

It has to be mentioned that some fabrication steps needed for clinical use, like color infiltration, polishing, and/or glazing, were omitted in this investigation. Previous studies on fracture strength of 3D-printed samples identified no significant effect of color infiltration, although stained specimens showed slightly lower biaxial strength values compared to unstained ones [24]. Sugiki et al. investigated the effect of staining on zirconia strength and found no significant reduction in strength of 3Y-TZP for most tooth-colored staining liquids. Only for liquids containing oxides of metals with the potential to change the crystal structure (Y, Er), a degradation could be observed [25]. For brittle materials like zirconia, surface polishing can increase fracture resistance if the fracture originates from the FDP surface, but there is also the danger that flaws induced into the surface due to improper execution may as well have a contrary effect. Amaral et al. found no such negative effects regarding the fatigue limit of 3-unit zirconia FDPs due to adjustments by grinding at the basal sides of the connectors [26].

The individual internal crown cavities used in this investigation led to slight deviations in FDP geometry within each test group. Distance measurement between the aligned individual surfaces revealed a mean absolute distance deviation between 10 and 20 µm and maximum absolute deviations along the whole internal surfaces between 30 and 70 µm. These deviations can be considered negligibly small in relation to the investigated FDP wall thicknesses. Cemented FDPs had also been checked for correct orientation before mechanical loading. The loaded cusps showed—with FDPs tilted by 30° in the buccal direction—angular deviations from the horizontal plane less than 2°. The position of the load application point during chewing simulation given by the wear facet on the loaded cusp was compared to the planned loading position. Predefined thresholds of 0.2 mm for mean deviation and 0.5 for maximum deviation between planned and real were not exceeded. Thus, aging and fracture load tests were well standardized. The use of a steel indenter had no effect on the fracture load determined for the milled FDPs with fractures originating at or near the connectors far from the loading site. With the 3D-printed FDPs, however, fracture originated near the loading site, and higher fracture loads could be expected for antagonists made of materials with a lower Young’s modulus (composite resin, enamel, titanium, glass ceramics). Since steel has about the same Young’s modulus as CrCo alloy and zirconia, this test condition has clinical relevance and states a worst-case scenario. The same is true for the investigated load case with an eccentric test force tilted by 30°, and the chewing simulation with a magnitude of 108 N chosen for the posterior region was meant to simulate at least 5 years of clinical use.

Based on the findings of this study, it can be assumed that it would be possible to achieve similar fracture resistance for 3D-printed zirconia FDPs compared to milled FDPs with an optimized pontic design or massive pontics and consequently longer firing times. The drawback of DLP or SLA zirconia printing compared to milling, up to date, is the higher fabrication time. Therefore, some research groups are dealing with ultra-fast debinding methods [27]. However, besides promising results for some showcase objects, it remains unclear if high demands for dental restorations with regard to material strength and accuracy can be met for dental restorations. Advantages of the 3D-printing technology are the possibility to create structured surfaces with the potential of enhanced bond strength [28,29] as well as to fabricate restorations without radius correction at recessed corners as given for tooth preparations including grooves or boxes. Both options would be advantageous for the fabrication of wing-retained resin-bonded FDPs. Despite these promising results and the excellent clinical performance of milled monolithic or vestibularly veneered zirconia restorations in the last years [30,31], more in vitro studies on the fracture resistance and fit of 3D-printed short-span zirconia FDPs dealing with different and possibly more challenging situations should be carried out to eliminate shortcomings before the start of the first clinical trials.

Individual calibration was a strength of this investigation, leading to a better fit of the printed restorations. Especially in the vertical direction, with assessed individual scaling factors S_z,indiv_ ≈ 1.36 being clearly higher than the universal scaling factor S_z,univ_ = 1.34, crowns would have been fabricated too short. With a crown height of about h = 8 mm, the theoretical discrepancy when printing zirconia FDPs with universal scaling factors would have been h (S_z,univ_/S_z,indiv_ − 1) = −118 µm. In the horizontal direction, deviations between universal and individual scaling factors were much smaller, but individual scaling parameters were not constant over the investigated time span of one and a half years. This is not surprising since with milling, individual scaling factors are provided for each zirconia batch.

## 5. Conclusions

Within the limitations of this laboratory study providing information about the performance of minimally invasive 3Y-TZP FDPs, all restorations showed acceptable fracture resistances with regard to clinical use for all wall thicknesses irrespective of the fabrication technology. However, the milled FDPs with greater wall thickness outperformed their 3D-printed counterparts, giving the dentist a higher safety margin. This gap may be closed for 3D-printed zirconia FDPs by geometry optimization of the hollowed-out pontics. Finite element analyses as well as further laboratory studies with promising modified geometries should clarify this issue.

Variability of individual scaling factors determined in this investigation and their deviation from the universal scaling factors provided by the manufacturer indicated the need for such parameters, in particular for FDPs with larger dimensions.

## Figures and Tables

**Table 1:** Firing protocol used for debinding and presintering of 3D-printed zirconia samples.

Start Temperature [°C]	End Temperature [°C]	Time [hh:mm]
0	150	00:15
150	200	00:10
200	320	20:00
320	320	01:00
329	490	05:40
490	490	01:00
490	1100	01:01
1100	100	01:00
	Total time	30:06

**Table 2:** Final sintering procedure used with presintered samples.

Start Temperature [°C]	End Temperature [°C]	Time [hh:mm]
0	1200	02:00
1200	1200	01:00
1200	1500	01:00
1500	1500	02:00
1500	0	01:20
	Total time	07:20

## Data Availability

The original contributions presented in the study are included in the article, further inquiries can be directed to the corresponding author.
